# An Audit of the DNACPR Policy at Malta's Mount Carmel Hospital

**DOI:** 10.1192/bjo.2022.499

**Published:** 2022-06-20

**Authors:** Sean Warwicker, Annalise Bellizzi

**Affiliations:** Mount Carmel Hospital, Attard, Malta

## Abstract

**Aims:**

A consideration for patient dignity in end-of-life care dictates that good clinical judgment should be exercised in advance resuscitation decisions. The COVID-19 pandemic, and its inherent risks to caregivers, only adds to this importance. Our aim was to audit the standards for the DNACPR policy at Mount Carmel Hospital (MCH), which is Malta's major inpatient psychiatric hospital, against those at Saint Vincent De Paule Residence (SVPR), which is a long-term care facility where DNACPR decisions are taken by geriatricians as opposed to psychiatrists.

**Methods:**

Resuscitation status designation and rates of form completion were measured in the five chronic psychiatric inpatient wards at MCH. This 98-patient population was compared against an age-matched cohort from SVPR to evaluate differences in decision-making.

Medical comorbidities and frailty scores (measured using the Clinical Frailty Scale) were compared between the two groups. As far as age-groups would allow, as many patients with a psychiatric comorbidity as possible were included from SVPR (36).

Z-score testing for two population proportions was used to evaluate the differences in resuscitation status designation. The Independent Sample T-Test was used to compare means in medical comorbidity and frailty. A p-value of <0.05 was used to assume statistical significance.

**Results:**

Rates of resuscitation form completion were 73.47% and 94.90% in MCH and SVPR, respectively. In those patients with completed documentation, 9.72% of patients were designated as “Not for CPR” in MCH, compared to 61.29% in SVPR.

Between these two age-matched cohorts, the mean frailty score was slightly greater in SVPR, which was not statistically significant (5.83 vs 5.48, p = 0.1456). The mean number of medical comorbidities was significantly greater in the SVPR cohort (3.50 vs 2.47, p = 0.0002).

**Conclusion:**

This striking difference in DNACPR designation suggests that geriatricians have a higher threshold for determining whether a patient would benefit from CPR compared to psychiatrists. Furthermore, rates of resuscitation form completion at MCH were disappointing. The greater likelihood for chronic psychiatric inpatients to be designated “For CPR” may be due to the perception that this entails a higher level care. In reality, in older, frailer patients, CPR may only prolong suffering, while a “Not for CPR” decision does not necessarily imply an omission of care.

In Malta, we've tailored resuscitation training to the inpatient psychiatry setting, which includes stations on decision-making and COVID-19.

